# Killing Effect of *Bacillus velezensis* FZB42 on a *Xanthomonas campestris* pv. *Campestris* (Xcc) Strain Newly Isolated from Cabbage *Brassica oleracea* Convar. *Capitata* (L.): A Metabolomic Study

**DOI:** 10.3390/microorganisms9071410

**Published:** 2021-06-29

**Authors:** Hynek Mácha, Helena Marešová, Tereza Juříková, Magdaléna Švecová, Oldřich Benada, Anton Škríba, Miroslav Baránek, Čeněk Novotný, Andrea Palyzová

**Affiliations:** 1 Institute of Microbiology of the Czech Academy of Sciences, Vídeňská 1083, 142 20 Prague, Czech Republic; hynek.macha@biomed.cas.cz (H.M.); maresova@biomed.cas.cz (H.M.); tereza.jurikova@biomed.cas.cz (T.J.); majdulas@seznam.cz (M.Š.); benada@biomed.cas.cz (O.B.); anton.skriba@biomed.cas.cz (A.Š.); novotny@biomed.cas.cz (Č.N.); 2 Department of Analytical Chemistry, Faculty of Science, Palacký University, 17. Listopadu 12, 771 46 Olomouc, Czech Republic; 3 Faculty of Horticulture-Mendeleum, Mendel University, Valtická 337, 69144 Lednice, Czech Republic; baranek@mendelu.cz

**Keywords:** *Xanthomonas campestris* pv. *campestris*, *Bacillus velezensis* FZB42, antagonism, cyclic lipopeptides, siderophore, killing effect, metabolomic analysis

## Abstract

The potential use of *Bacillus velezensis* FZB42 for biological control of various phytopathogens has been documented over the past few years, but its antagonistic interactions with xanthomonads has not been studied in detail. Novel aspects in this study consist of close observation of the death of *Xanthomonas campestris* pv. *campestris* cells in a co-culture with *B. velezensis* FZB42, and quantification of lipopeptides and a siderophore, bacillibactin, involved in the killing process. A new robust Xcc-SU isolate tolerating high concentrations of ferric ions was used. In a co-culture with the antagonist, the population of Xcc-SU was entirely destroyed within 24–48 h, depending on the number of antagonist cells used for inoculation. No inhibitory effect of Xcc-SU on *B. velezensis* was observed. Bacillibactin and lipopeptides (surfactin, fengycin, and bacillomycin) were present in the co-culture and the monoculture of *B. velezensis.* Except for bacillibactin, the maximum contents of lipopeptides were higher in the antagonist monoculture compared with the co-culture. Scanning electron microscopy showed that the death of Xcc-SU bacteria in co-culture was caused by cell lysis, leading to an enhanced occurrence of distorted cells and cell ghosts. Analysis by mass spectrometry showed four significant compounds, bacillibactin, surfactin, fengycin, and bacillomycin D amongst a total of 24 different forms detected in the co-culture supernatant: Different forms of surfactin and fengycin with variations in their side-chain length were also detected. These results demonstrate the ability of *B. velezensis* FZB42 to act as a potent antagonistic strain against Xcc.

## 1. Introduction

The bacterial genus *Xanthomonas* includes phytopathogens that can be encountered worldwide and cause diseases of various economically important vegetable species. The bacterial species *Xanthomonas campestris* pv. *campestris* (Xcc) causes black rot in cruciferous vegetables, a disease responsible for severe economic losses [1]. Generally, the control of infections is mainly achieved with chemical pesticides, less susceptible plant varieties, and by destroying the contaminated plants. Biological control is an ecological-friendly alternative, less toxic for non-target species, and with the potential to suppress resistant pathogens [2,3].

*Bacillus* spp. is a group of bacteria widely used in biocontrol research, and commercial formulations are available on the biopesticide market [4]. Several strains of the genus *Bacillus* exhibit biological control activity against plant pathogens, including Xcc [1,5]. *B. amyloliquefaciens* strains were reported to antagonize *X. citri* sbsp. *citri* [6], *X. axonopodis* pv. *dieffenbachiae* [7], and *X. campestris* pv. *campestris* [8,9]. *B. velezensis* strain FZB42 (formerly *B. amyloliquefaciens* subsp. *plantarum* FZB42) has become the Gram-positive prototype for biocontrol of plant pathogens [4].

Almost 10% of the *B. velezensis* FZB42 genome is involved in the synthesis of antimicrobial compounds, the principal ones being the lipopeptides surfactin, bacillomycin D, and fengycin [10]; and polyketides bacillaene, macrolactin, and difficidin [11]. The production of bioactive and surfactant compounds occurs during the late growth phase and can be stimulated by plant pathogens [12,13]. All secondary metabolites produced by *B. velezensis* FZB42 are summarized in the review of Fan and coworkers [4]. Studies in vitro suggest that the antifungal activity of this bacterium is mainly due to the synthesis of the cyclic lipopeptides fengycin and bacillomycin D, whereas antibacterial activity is due to the production of polyketides, bacilysin, and bacteriocins. Difficidin, macrolactin, and bacillaene also possess antibacterial activity [14,15].

The cyclic lipopeptide surfactin, produced by many *Bacillus* species, is one of the most effective biosurfactants. As a molecule of amphiphilic nature, it destabilizes membranes and disturbs their integrity [16]. The antibacterial activity of surfactin inhibits the growth of bacterial pathogens, e.g., *Pseudomonas syringae* [17], *X. axonopodis* pv. *glycines* [18], *Klebsiella pneumoniae*, *Salmonella typhimurium*, *Staphylococcus aureus*, and *Escherichia coli* [19], or has a bactericidal effect, e.g., *Clostridium perfringens* [20], *Listeria monocytogenes*, *B. cereus,* and *S. aureus* [21]. Fengycin was demonstrated to have an inhibitory effect on fungal growth, e.g., on *Fusarium graminearum* [22], *F. moniliforme* [23], *Venturia ineaqualis* [24], or on conidia germination, e.g., *Podosphaera fusca* [25] and *Plasmopara viticola* [26]. Interestingly, the effect of fengycin was promoted in the presence of surfactin [24,26]. Fengycin also changes the hyphal morphology of *F. graminearum* [22]. Medeot and coworkers [27] reported that fengycins from *B. amyloliquefaciens* MEP218 also exhibited antibacterial activity by exerting a bactericidal effect on *X. axonopodis* pv. *vesicatoria* and *P. aeruginosa*.

The third major lipopeptide produced by *B. velezensis* FZB42, bacillomycin D, was identified as a potent antifungal metabolite against a spectrum of fungi [28]. It was reported to inhibit mycelial growth, sporulation, and spore germination of *Aspergillus flavus*, causing damage to the cell walls and membranes of the fungal hyphae and spores [29]. Similar effects of bacillomycin D isolated from *B. velezensis* HN-2 on the fungal phytopathogen *Colletotrichum gloeosporioides* were described by Jin and coworkers [30]. An interesting synergistic fungicidal activity of bacillomycin D, isolated from *B. subtilis* B38, with the polyene amphotericin B against *Candida albicans,* was also reported [31]. However, the adverse effects of bacillomycin are not limited to fungal organisms. Antibacterial activity of a bacillomycin D-like cyclic lipopeptide, bacillomycin DC, isolated from *B. amyloliquefaciens* HAB-2, against the bacterium *Burkholderia pseudomallei,* was reported. The compound inhibited growth of the pathogen and caused a roughening of the cell surface and damage to the cell membrane [32]. Another major non-ribosomal peptide, produced by *B. velezensis,* is bacillibactin, which acts as a catechol-type siderophore for the compartmentalization of iron in competition with other microbial cohabitants [14].

Xcc is a rod-shaped, non-sporulating, Gram-negative, motile bacterium with an optimum growth temperature of 25–30 °C. The Xcc infection of cruciferous crops is characterized by yellow V-shaped lesions, progressing from leaf margins to the center through vascular tissue, and by the darkening of veins resulting in wilting and leaf necrosis [33]. Xcc produces an α-hydroxycarboxylate-type siderophore named xanthoferrin that is required for growth under low-iron conditions and optimal virulence [34]. Generally, xanthomonads are believed not to produce secondary metabolites with antibiotic activity. Despite that, they are able to negatively influence other microorganisms cohabiting their environment, as is documented in the following examples. *X. campestris* pv. *vitians* was shown to reduce the bacterial population of lettuce-leaf microbiota [35]. In an antagonistic interaction with *B. thuringiensis* in a mixed culture, Xcc was able to interfere with cell division and sporulation of *B. thuringiensis* by producing diffusible signaling factors [36]. *X. perforans* was found to produce different bacteriocin-like compounds that were antagonistic toward its phytopathogenic competitor *X. euvesicatoria* [37]. *X. albilineans* was found to produce the pathotoxin albicidin which, in addition to its crucial role in pathogenesis and development of disease symptoms in the sugarcane plant host, also inhibits prokaryotic DNA replication and exhibits a bactericidal effect against a range of Gram-positive and Gram-negative bacteria [38].

Various *B. velezensis* strains were demonstrated to inhibit the growth of *X. campestris* pv. *campestris* in confrontation experiments on agar medium [2,39]. The purpose of our study was to analyze the inhibitory action of *B. velezensis* FZB42 on the growth of *X. campestris* pv. *campestris* under in vitro conditions in a dual liquid-medium culture and provide evidence of antagonist metabolites involved in the destruction of the Xcc population and the kinetics of bacterial elimination. A new strain to be used in our study, Xcc-SU, was isolated from a cabbage field massively attacked by an Xcc infection, and we will conduct an antagonistic study with a fresh Xcc isolate not affected by lengthy conservation. The biochemical and molecular genetic characteristics of the novel Xcc strain were obtained and used for its taxonomical classification. The conditions of the dual culture of *B. velezensis* FZB42 and Xcc-SU were optimized with respect to the proportion of the two microbes used for inoculation. The antagonistic interaction was investigated over time by measuring viable counts of the two microorganisms and carrying out the metabolomic analysis, including quantification of crucial secondary metabolites produced by *B. velezensis* FZB42.

## 2. Materials and Methods

### 2.1. Microorganisms and Growth Media

*Bacillus velezensis* FZB42 (DSM 23117) was obtained from DSMZ collection (Braunschweig, Germany). *Pseudomonas aeruginosa* PAO1 (ATCC 15692), *P. fluorescens* (ATCC 13525), and *P. putida* B10 (ATCC 39169) were purchased from the ATTC collection (Manasses, VA, USA), and *Pseudomonas* sp. DF was isolated from the activated sludge (Třeboň, wastewater treatment plant, Czech Republic) and classified at the Institute of Microbiology of the Czech Academy Sciences, Prague. The *Xanthomonas campestris* pv. *campestris* (Xcc) strains Xcc 3811, Xcc 1279A, and Xcc 3871A were purchased from the NCTC collection (University of Warwick, UK). The Xcc-SU strain was isolated from an infested cabbage field in north Bohemia (coordinates: 50.5872317N latitude, 15.0433481E longitude). The yeast *Kluyveromyces lactis* IFO 1267 (killer-toxin + strain) was a gift from the Department of Microbiology and Genetics, Charles University, Prague.

The media used for growth were Luria-Bertani broth (LB, g/L: 10 tryptone, 5 yeast extract, 10 NaCl, pH 7.0) or Bushnel-Haas medium (BH, g/L: 0.2 MgSO_4_·7H_2_O, 0.02 CaCl_2_·2H_2_O, 1 K_2_HPO_4_, 1 KH_2_PO_4_, 1 NH_4_NO_3_, 5 glucose, pH 7.0) containing a high (200 μM) or low (8 μM) Fe^3+^ concentration. For antagonistic studies, a mineral medium was used (M9, g/L: 0.5 NaCl, 3.0 KH_2_PO_4_, 14.62 Na_2_HPO_4_, and 1.0 NH_4_Cl, pH 7.0) supplemented with glucose (5 g/L) and trace elements (M9TE, mg/L: 200 MgSO_4_·7H_2_O, 10 CaCl_2_·2H_2_O, 2.16 FeCl_3_·6H_2_O). A selective culture medium MCS (mCS20ABN, Duchefa Biochemie, Haarlem, Netherlands) supplemented with bile salt No.3 (0.5 g/L, Difco) was used to identify Xcc strains in dual cultures. In order to solidify the medium for dual cultures (BH and MCS) or short-term strain maintenance (LB), 22 g/L of agar was added.

### 2.2. Isolation and Taxonomy of the Newly Isolated Xcc-SU Strain

Leaf samples of napa cabbage (*Brassica rapa* subsp. *pekinensis)* were cut into pieces 2 cm long and placed in a 500 mL flask containing 50 mL of LB medium. The content was shaken for 4 h at 28 °C, and the isolated bacteria were spread by serial dilution in LB medium onto Petri plates. The plates were incubated at 28 °C for 48 h. Monocolony isolates were picked and maintained for biochemical and genetic studies. Molecular identification was carried out by sequencing the 16S rRNA gene amplified by polymerase chain reaction using a universal primer set (Fwd27 and Rev1492). Total DNA was isolated from the bacterium using a QIAamp DNA mini kit (Qiagen, Düsseldorf, Germany). PCR amplicons were purified using a High Pure PCR Product Purification Kit (Roche, Basel, Switzerland), following the manufacturer’s instructions. The nucleotide sequences were determined by sequencing on an ABI PRISM 3130xl Genetic Analyzer (Applied Biosystems, Waltham, MA, USA), edited by Chromas Lite software (Technelysium Pty Ltd., Brisbane, Australia), and assembled using SeqMan (DNASTAR, Inc., Madison, WI, USA). The search for 16S rRNA gene sequence similarity was performed using the GenBank data library and the BLASTN program (NCBI, Bethesda, MD, USA). Physiological and biochemical characterizations were carried out by the Czech Collection of Microorganisms, Masaryk University, Brno.

### 2.3. Antagonism Assays on Solid Medium

The antagonistic effects of *B. velezensis* FZB42, *K. lactis* IFO 1267, *P. aeruginosa* PAO1, *P. fluorescens*, *P. putida* B10, and *Pseudomonas* sp. DF on the collection of Xcc strains 3811, 1279A, and 3871A, and on the natural isolate, Xcc-SU, were measured using the dual-culture technique on BH agar medium. Xcc was spread on the agar surface, and, simultaneously, the antagonist bacterium was inoculated at the center of the plate using a drop (10 μL) of a fresh culture (10^8^ CFU/mL) grown in LB medium. Monoculture BH plates inoculated with either the antagonist or the pathogenic strain served as the controls. All plates were incubated at 28 °C for 7 days. The antagonistic effect was visualized as a halo zone formed around the antagonist colony. The experiments were carried out in three replicates.

### 2.4. Antagonism Assay in Liquid-Medium Culture

Growth inhibition of Xcc-SU was tested in a dual culture with *B. velezensis* FZB42 in liquid M9TE medium. The inoculum was prepared in two stages. The primary inoculum of each bacterium was obtained by inoculating 100 mL of LB medium with a vial (1 mL) of a glycerol-stock bacterial culture and subsequent incubation on an orbital shaker (200 rpm) for 24 h at 28 °C. The overnight culture was centrifuged, washed, and resuspended in M9 medium and then inoculated into 100 mL of M9TE medium and incubated at 28 °C for 24 h. The fresh bacterial suspensions of *B. velezensis* and Xcc-SU, both harvested in the exponential growth phase and adjusted to a final concentration of 10^8^ CFU/mL, were used for inoculation of the dual culture using respective ratios of 1:3, 1:15, or 1:100 CFU/mL. The total volume of the dual culture was 100 mL of M9TE medium and was incubated into a 500-mL culture flask and agitated on an orbital shaker (200 rpm) at 28 °C. A monoculture of each strain, incubated under similar conditions, was used as the control. Samples were collected during growth to measure the culture density as absorbance at 600 nm (A_600_), pH, and colony-forming units (CFU) of both microorganisms. The samples used for the metabolomic analysis were centrifuged to obtain the supernatant, and this was lyophilized before analysis. All experiments were conducted in duplicate.

### 2.5. Scanning Electron Microscopy Analysis

Samples removed from the dual culture were fixed (3% glutaraldehyde, 0.1 M cacodylate buffer, pH 7.4) for 48–96 h at 4 °C. They were centrifuged (14,300× *g*, 5 min, room temperature), and the sediment was resuspended in 500 µL of 3% glutaraldehyde and fixed overnight. After fixation, the samples were centrifuged again (12,000× *g*, 5 min, room temperature) and washed with cacodylate buffer (20 min, three times). The cells were sedimented onto poly-_L_-lysine-treated glass coverslips for 24 h at 4 °C. The coverslips were then washed with double-distilled water at room temperature, post-fixed with 1% osmium tetroxide for 1 h, washed again with ddH_2_O (20 min, three times), dehydrated in a graded ethanol series and absolute acetone, and critical-point dried (K850, Quorum Technologies Ltd., Ringmer, UK). The dried coverslips were mounted onto aluminum specimen stubs using Wire Glue (Anders Products, Andover, MA, USA) and sputter-coated with 3 nm of platinum in a high-resolution Turbo-Pumped Sputter Coater Q150T (Quorum Technologies Ltd., Ringmer, UK). The samples were examined using an FEI Nova NanoSEM scanning electron microscope (FEI, Brno, Czech Republic) at 3 or 5 kV using Everhart–Thornley Detector (ETD), Circular Backscatter Detector (CBS), and Through the Lens Detector (TLD).

### 2.6. Metabolite Extraction

Culture samples were removed at 12-h intervals. The supernatants were obtained by centrifugation (10,000× *g*, 10 min, 4 °C), decanting, and subsequent lyophilization. The lyophilized supernatants were dissolved in 50% LC-MS-grade acetonitrile (Honeywell CHROMASOLV™, VWR, Stříbrná Skalice, Czech Republic) to a final concentration of 1 mg/mL. A volume of 100 µL of the dissolved sample was then mixed with 400 µL of methanol (Honeywell CHROMASOLV™, VWR, Stříbrná Skalice, Czech Republic) and deep-frozen (30 min, -80 °C). The precipitates were removed by centrifugation (14,000× *g*, 10 min, 4 °C), and the supernatants were transferred to a new microcentrifuge vial and dried using a Savant SpeedVac vacuum concentrator (Thermo Scientific, Waltham, MA, USA) for 2 h at 35 °C. The dried samples were dissolved in 100 µL of 50% LC-MS-grade acetonitrile.

### 2.7. Metabolite Separation, Identification, and Quantitation

Samples were analyzed by high-performance liquid chromatography-mass spectrometry (HPLC-MS) using the external calibration curve method. Surfactin, fengycin, and bacillomycin were quantified using a Waters Acquity M-class HPLC system connected to a Synapt G2-Si Q-TOF mass spectrometer (Waters Corporation, Manchester, UK). Each sample (1 µL) was injected in triplicate onto an Acquity HSS T3 C18 analytical column (1.8 µm, 1.0 × 150 mm, Waters Corporation, Manchester, UK) kept at 40 °C. Analytes were separated using A/B solvent mixture with gradient elution (flow rate 50 µL/min) under the following conditions: 0 min (5% B); 2 min (5% B); 7 min (99% B); 14 min (99% B); 14.5 min (5% B); and 20 min (5% B). Solvent A contained 0.1% formic acid water solution, and solvent B contained 0.1% formic acid in acetonitrile. 

Higher sensitivity was needed for bacillibactin quantification. Therefore, a Dionex UltiMate 3000 HPLC system (Thermo Fisher Scientific, Waltham, MA, USA) connected to a SolariX 12T Fourier transform ion cyclotron resonance mass spectrometer (Bruker Daltonik, Bremen, Germany) was used. Samples (2 µL) were injected on the same Acquity column in triplicate. Gradient elution of bacillibactin was performed at 50 µL/min flow rate: 0 min (2% B); 2 min (2% B); 9 min (60% B); 11 min (99% B); 14 min (99% B); 14.5 min (2% B); 20 min (2% B). Solvent A contained 0.1% formic acid and 1% acetonitrile in water, and solvent B contained 0.1% formic acid and 5% water in acetonitrile. 

Both mass spectrometers were operated in positive-ion mode. Ions were generated by electrospray ionization and the ion transfer optics were tuned to the highest intensity of standards. The mass spectra were collected in the 500–1600 *m/z* window with mass accuracy better than 50 ppm for Synapt and the 500–1500 *m/z* window with mass error better than 5 ppm for Solarix, respectively. All metabolites were identified by a combination of retention time, precise mass-to-charge ratio, and collision-induced dissociation experiments. Qualitative and quantitative data processing was performed by CycloBranch [40] version 2.0.8 (Prague, Czech Republic), Waters MassLynx 4.1 (Manchester, UK), and Bruker DataAnalysis 5.0 software (Bremen, Germany).

Surfactin and fengycin standards were obtained from Sigma-Aldrich (Czech Republic). Bacillibactin was purchased from EMC Microcollections (Tübingen, Germany). The external matrix match calibration curves were prepared from lyophilized growth medium spiked with commercial standards. Surfactin and fengycin standards were represented by several forms with variations in aliphatic side-chains, therefore, the sums of all forms were used in the construction of calibration curves, including the limit of detection (LOD) and the limit of quantitation (LOQ). Semi-quantification of bacillomycin D was performed based on the fengycin standard calibration curve. The LOD and LOQ for bacillibactin, surfactin and fengycin were defined as the sum of the background average with 3 (LOD) and 10 (LOQ) multiples of standard deviation. The LOD and LOQ calculated from the bacillibactin, surfactin, and fengycin signal-to-noise ratios were 1.9 and 4.5 ng/mL; 1.1 and 3.2 ng/mL; and 1.6 and 4.1 ng/mL, respectively.

## 3. Results and Discussion

### 3.1. Characterization and Identification of Xcc-SU

Out of eight monocolonies, cabbage-leaf isolates of the same morphological appearance were grown on LB agar medium and a single colony was selected and designated as strain Xcc- SU. It was characterized to be a Gram-negative, rod-shaped (in irregular clusters) aerobic bacterium. The colonies grown on nutrient Tryptic soy agar were circular, smooth, and glossy with a raised center. The optimum temperature for growth was in the range of 20–30 °C. The biochemical characteristics included positive biochemical results: catalase, oxidase; growth in Simons citrate; hydrolysis of gelatin, starch, Tween 80, aesculin, casein, and lecithin; and production of acids from mannitol, glucose, fructose, and xylose. Negative results: acid fermentation from glucose; production of fluorescein, arginine dihydrolase; hydrolysis of tyrosine; decarboxylation of tyrosine and ornithine; and growth in the presence of 6.5% NaCl. The molecular classification of Xcc-SU was carried out using 1.5 kb of the 16S rRNA sequence, which was amplified from the total DNA of the strain. The BLAST search showed that the strain belonged to the family *Xanthomonadaceae* and the genus *Xanthomonas*. The partial nucleotide sequence of 16S rRNA region (about 1350 nucleotides) showed a homologous identity (over 99%) with *X. campestris* pv. *campestris* (GenBank accession number NC_003902.1). The strain was designated as *Xanthomonas campestris* pv. *campestris* SU and the sequence of the 16S rRNA gene was deposited in the GenBank database under accession number MZ182354. 

### 3.2. Characterization of Xcc Strains and Dual-Culture Agar Diffusion Bioassay

Properties of Xcc strains obtained from the NCTC collection and the newly isolated Xcc-SU strain were compared by testing their growth in BH medium pH 7 containing glucose as the carbon and energy source and at high (200 μM) or low (8 μM) Fe^3+^ concentrations. Iron concentration is a factor that contributes to antagonistic interactions between microbes due to the competitive actions of siderophores [12,41], so we tested Xcc strains for their Fe^3+^ dependence. 

**Figure 1:**
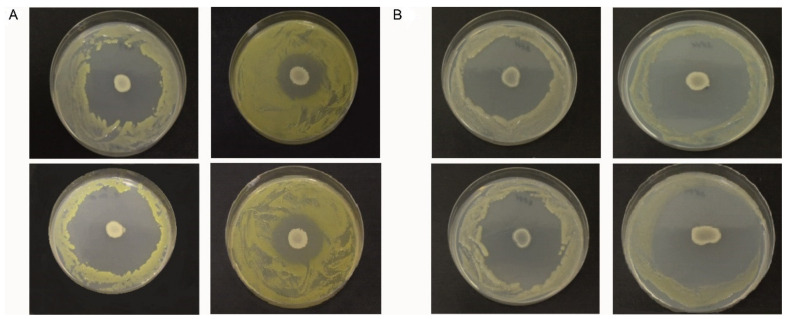
Inhibition of growth of Xcc strains by *B. velezensis* FZB42 on BH agar medium at 8 μM Fe^3+^ (left column) and 200 μM Fe^3+^ (right column): (**A**) top, Xcc 1279A; bottom, Xcc SU; (**B**) top, Xcc 3811; bottom, Xcc 3871A.

**Figure 2:**
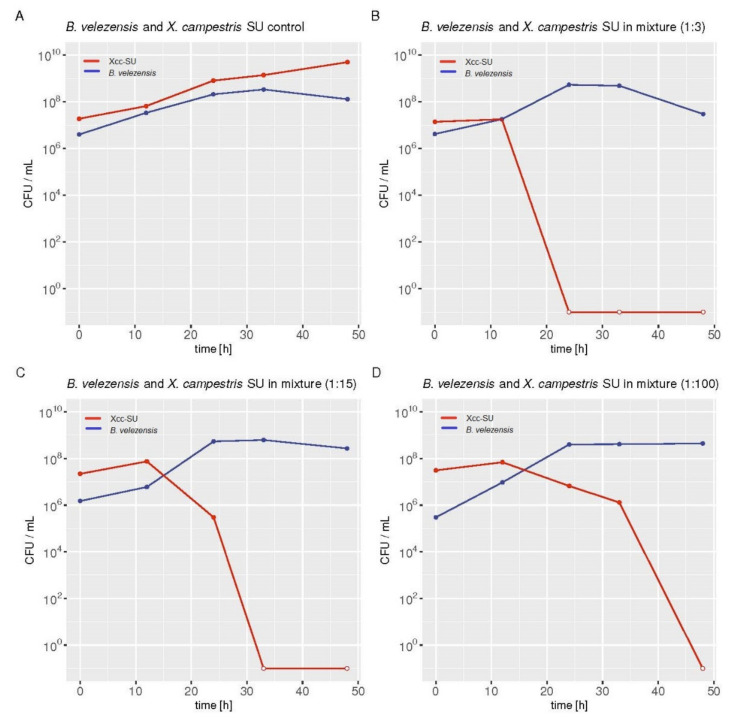
Xcc-SU dying rates in dual cultures with *B. velezensis* FZB42 grown in liquid medium. The ratios of *B. velezensis* to *X. campestris* SU at the time of inoculation was 1:3 (**B**), 1:15 (**C**), and 1:100 (**D**) in CFU/mL. (**A**) Representative growth curves of both microorganisms in control monocultures. The individual values in the graphs show the arithmetic means of CFU/mL measured at a given time in two independent cultures. The open dots in the Xcc plots indicate that no living Xcc bacteria were detected in the dual culture. R Core Team (2021) [46] free software environment for statistical computing and graphics was used for graphical presentation of the data. *B. velezensis* FZB42, blue line; Xcc-SU, red line.

Strains Xcc-1279A and Xcc-SU showed smaller growth inhibition zones when exposed to *B. velezensis* at 200 μM, compared to 8 μM Fe^3+^ (Figure 1A), which indicated a smaller antagonistic effect at the high iron concentration. In the case of Xcc-3871A and Xcc-3811 strains (Figure 1B), a similar size of inhibition zones was observed at both Fe^3+^ concentrations. As the siderophore bacillibactin was produced by *B. velezensis* FZB42 (see Figure 2), the results may indicate an active role of bacillibactin in the suppression of Xcc growth in solid medium by making Fe^3+^ ions unavailable to Xcc. In the case of the abundance of Fe^3+^ at 200 μM, the production of bacillibactin was reduced [42], and the effect of the siderophore was much less important. Mostly, the lipopeptides surfactin, fengycin, and bacillomycin (see Figure 2) were responsible for the inhibition of Xcc growth. High xanthan production has been shown to play a role in the antibiotic resistance of Xcc, and this effect could take place at the high Fe^3+^ concentration [43,44]. We can hypothesize that strains Xcc 3811 and Xcc 3871A produced less xanthan material than strains Xcc 1279A and Xcc SU, resulting in a less efficient protection against the lipopeptides of the antagonist, which could explain the different sizes of the inhibition zones observed with the two groups of Xcc strains at 200 μM Fe^3+^ [43,45].

When pseudomonas strains were used to inhibit Xcc under similar conditions, the inhibitory effect was extremely weak, with *P. aeruginosa* and *P. fluorescens* slightly inhibiting Xcc-1279A or Xcc-SU and *Pseudomonas* sp. DF1 inhibiting strains Xcc-3811 or Xcc-3871A. No inhibition was observed with *P. putida* and the yeast *K. lactis* IFO 1267. The Xcc-SU strain was chosen for the biocontrol metabolomic study with *B. velezensis* FZB42, because it was a phytopathogenic strain freshly isolated from a field massively struck by bacterial infection and exhibiting robust growth.

### 3.3. Microbial Interaction in the Dual Liquid Medium Culture of B. velezensis FZB42 and Xcc-SU

The inhibitory effect of *B. velezensis* FZB42 on the phytopathogen was studied in M9TE medium supplemented with glucose as a source of carbon and energy at a low Fe^3+^ concentration of 8 μM. To study the antagonistic effect, the low iron concentration was chosen based on the previous inhibition analyses. The doubling times of *B. velezensis* FZB42 and Xcc-SU in M9TE medium were 3 and 6 h, respectively. In order to follow the inhibitory effect in batch cultivation over time, the proportion of both microorganisms used for inoculation had to be optimized. The overnight inocula of both microorganisms were used to inoculate single and mixed cultures of *B. velezensis* with Xcc-SU at three different ratios (1:3, 1:15, and 1:100 CFU/mL). Figure 2 documents the time course of the killing effect of *B. velezensis* on Xcc-SU in the different inoculation ratios of *B. velezensis* to Xcc-SU. Killing was proportional to the ratios of the organisms at inoculation. When the concentration of *B. velezensis* was 100 times lower than that of Xcc-SU, the population of Xcc was eliminated within 48 h. When the antagonistic culture was only three times lower, Xcc was not detectable in the culture after a period of 24 h. The ratio of *B. velezensis*/Xcc-SU of 1:100 CFU/mL was chosen as the most suitable for further study.

In the dual culture, *B. velezensis* FZB42 achieved a total growth yield similar to that in its monoculture within 24 h. The number of viable cells did not decline until the end of cultivation. The monoculture of *B. velezensis* reached its maximum specific growth rate of 0.14 ± 0.003 h^−1^ within 24 h. No inhibitory effect of Xcc-SU on *B. velezensis* was observed. In the control monoculture, Xcc-SU reached a specific growth rate of 0.12 ± 0.003 h^−1^ and maximal growth yield approaching 5.0 ± 0.2 × 10^9^ within 48 h. A two-fold decrease in the number of living cells of Xcc-SU was observed when the incubation continued until 96 h.

### 3.4. Detailed Analysis of Biocontrol Metabolites Produced by B. velezensis FZB42 in the Dual Culture with Xcc-SU

**Figure 3:**
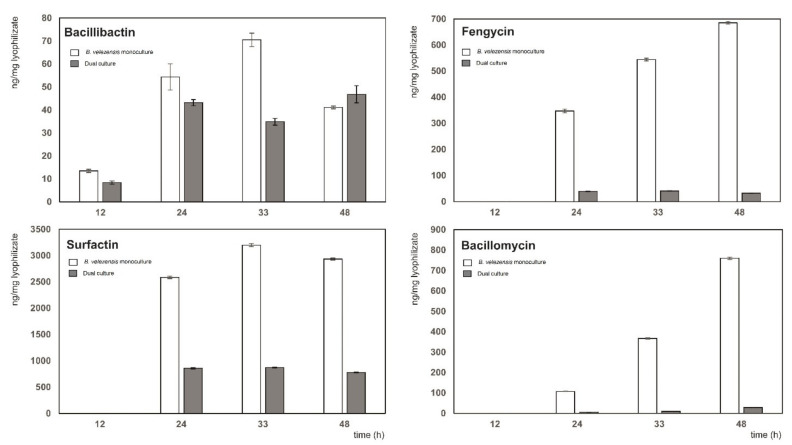
Production of biocontrol metabolites in a dual culture of *B. velezensis* FZB42 and Xcc-SU grown in liquid medium. The ratio of *B. velezensis* to Xcc-SU at the time of inoculation was 1:100 (CFU/mL). Dual culture, full columns; *B. velezensis* monoculture, empty columns.

The MS analysis revealed four compounds, bacillibactin, surfactin, fengycin, and bacillomycin D, in 24 different forms (Supplementary Table S1) in the cell-free supernatant. The biocontrol siderophore and lipopeptide metabolites were present in both the dual culture and in the monoculture of *B. velezensis* (Figure 3). All metabolites were present until at least the 48th hour of cultivation so that they could contribute to the annihilation of the Xcc-SU population (cf. Figure 2). The observed bactericidal effect is in accordance with the antagonistic studies where various *Bacillus* antagonists were used for the biological control of bacterial and fungal phytopathogens. For instance, the bactericidal effect of fengycins from *B. amyloliquefaciens* MEP218 on *X. axonopodis* pv. *vesicatoria* and *P. aeruginosa* PA01 was documented by Medeot et al. [27]. Our analyses showed that the 13 ionic forms belong to the lipopeptide fengycin (Supplementary Table S1). Using MS/MS analysis of the doubly charged ion with an *m/z* 732.406 (4A,4B in Supplementary Figure S1), we identified fengycin A with a C16 side-chain. Other forms with variations in the side-chain were confirmed based on the same retention time as their standards or an exact mass. In addition to the metabolite mentioned above, the other compound with an *m/z* 1036.697 (2A,2B in Supplementary Figure S1) was detected and identified as the lipopeptide surfactin with a C12 side-chain. Based on the same retention time with the surfactin standard, we confirmed the presence of other forms with variations in side-chain length (Supplementary Table S1). Interestingly, growth-inhibitory and morphology-modification effects induced by a combination of fengycin and surfactin on the ascomycete fungus *Venturia inaequalis* were observed, suggesting a possible synergistic effect [24,47]. Similarly, both surfactin and fengycin contributed to the activity of *B. subtilis* supernatant against the oomycete *Plasmopara viticola* [26]. Further, our data showed several signals in dual cultures associated with the presence of bacillomycin D. Using MS/MS, we identified the ion with *m/z* 1031.548 (1A,1B in Supplementary Figure S1) as bacillomycin D with a C14 side-chain. Other homologs were confirmed by exact masses. Antibacterial activity of bacillomycin isolated from *B. amyloliquefaciens* against *Burkholderia pseudomallei*, including growth inhibition and cell membrane damage, was reported [32]. The lipopeptides measured in the dual- and single cultures of *B. velezensis* FZB42 were not present in the monoculture of Xcc-SU. Polyketides such as bacillaene, macrolactin, or difficidin, known to have antibacterial activity [15,48], were not detected in the dual culture of *B. velezensis* with Xcc-SU nor in the monoculture of the antagonist. In addition, using MS/MS spectra, we identified a dominant protonated form of the compound with *m/z* 883.263 (3A,3B in Supplementary Figure S1), which was identified as bacillibactin. It mediates iron transport in *Bacillus subtilis* [49] and is involved in antibacterial or antifungal activities [50].

In addition to the qualitative analysis of the cell-free extracts of dual cultures, a quantitative analysis of individual metabolites was performed. The quantity of lipopeptides (ng per mg of lyophilized supernatant) measured in the monoculture of *B. velezensis* FZB42 was much higher than those in the dual culture except for the siderophore bacillibactin, where the maximum content in both cultures was comparable. The maximum amount of bacillibactin, surfactin, and fengycin in the dual culture was achieved in 24 h, whereas bacillomycin increased during cultivation up to 48 h (Figure 3). In the monoculture of *B. velezensis* FZB42, the maximum contents of surfactin (3198 ± 30 ng/mg) and bacillibactin (70 ± 3 ng/mg) were reached at 33 h, whereas those of fengycin (685 ± 5 ng/mg) and bacillomycin (760 ± 6 ng/mg) at 48 h. The maximum amounts of bacillibactin, surfactin, fengycin, and bacillomycin detected in the dual culture were about 1.4, 3.8, 14.0, and 26.7 times lower (Figure 3).

The presence of Xcc-SU in the co-culture with *B. velezensis* FZB42 did not enhance the production of lipopeptides, as observed for the production of iturin, fengycin, and bacillibactin by *B. amyloliquefaciens* in the presence of some phytopathogens [12,13]. In contrast, the production of fengycin, surfactin, and bacillomycin in the dual culture was considerably reduced compared to the monoculture of *B. velezensis* (Figure 3). In spite of that, the maximal surfactin concentration of 46 ± 3 µg/mL detected in the dual culture was similar to that of 50 µg/mL reported by Chen et al. [51], exhibiting the bacteriostatic effect. The other two lipopeptides, fengycin and bacillomycin, were produced in the dual culture at respective maximal concentrations of 2.0 ± 0.4 µg/mL and 1.6 ± 0.3 µg/mL. Purified bacillomycin and fengycin lipopeptides were shown to exhibit the antibiotic activity in the range of concentrations of 2–32 µg/mL and 6.25 µg/mL, respectively [27,52]. The reason for low concentration of metabolites could be a competition for nutrients between the two microorganisms during growth. The optimum nitrogen concentration reported for *B. amyloliquefaciens*, with respect to the synthesis of lipopeptides, is about 10 times higher than that in our M9 medium [53], so competition could primarily concern assimilable nitrogen. In streptomycetes, carbon and nitrogen source limitation resulted in a significant reduction in antibiotic synthesis [54]. Similarly, in *B. amyloliquefaciens*, indirect evidence of the relationship between the provision of sufficient quantities of building precursors and the overproduction of surfactin was reported [55]. Another factor contributing to the reduction in the synthesis of lipopeptides could be a relative lack of iron caused by competition of both microorganisms for Fe^3+^ that was present at a low concentration of 8 µM in the dual culture. Such a scarcity of Fe^3+^ could result in a decrease in lipopeptide synthesis since iron serves as a cofactor for enzymes involved in the production of lipopeptides [56,57].

As for the production of bacillibactin, there was little or no reduction in its synthesis in the dual culture, possibly because its total level was approximately 10 times lower than that of each of the other three lipopeptides produced under nonlimiting conditions in the monoculture of *B. velezensis*. Consequently, its biosynthesis could be less affected by a limit in carbon and nitrogen supply (Figure 3). In addition, at a low Fe^3+^ concentration of 8 µM used in the medium, the Fur regulon-controlling bacterial iron homeostasis was derepressed, which probably led to the non-repressed synthesis of bacillibactin [42].

A possibility exists that the level of lipopeptides in the dual culture could also be reduced by biodegradative activity of Xcc-SU. Xanthomonads have been shown to degrade various recalcitrant compounds such as the herbicides chlopyrifos and lindane [58,59] or hydrocarbons [60]. Xcc also possesses extracellular proteases [61]. However, experiments with *B. mojavensis* demonstrated that a lipopeptide mixture produced by this microorganism was relatively insensitive to the action of proteolytic enzymes [62]. Also, the fact that the amount of bacillibactin was not significantly reduced in the dual culture, despite the chemical similarity with the other lipopeptide molecules, seemed not to support this hypothesis.

### 3.5. Morphology Changes of Xcc Cells in the Presence of B. velezensis FZB42

**Figure 4:**
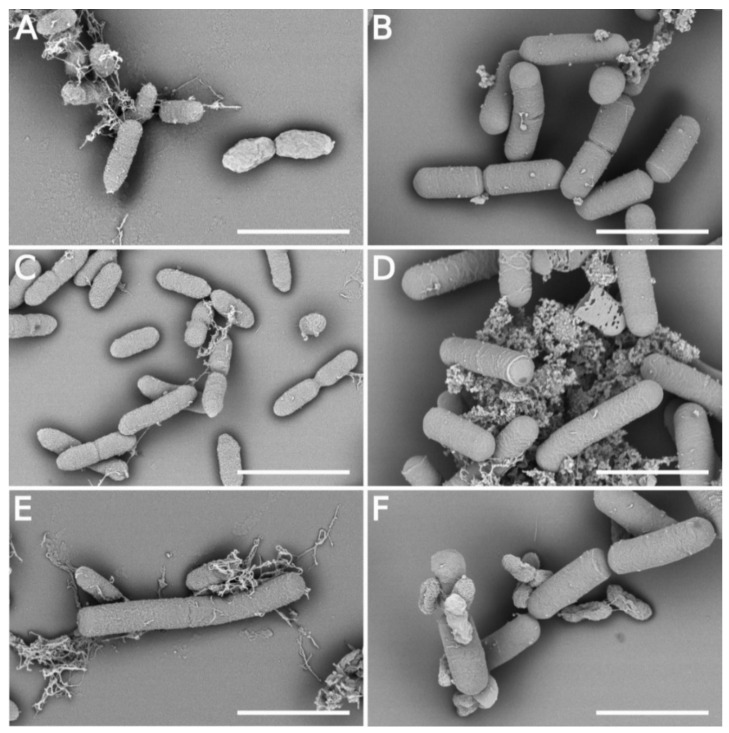
Time course of the interaction of *B. velezensis FZB42* and Xcc-SU growing in a dual culture visualized by SEM (scale 2 μm): (**A**) start inoculation: Xcc-SU monoculture, (**B**) start inoculation: *B. velezensis* monoculture, (**C**) 48 h after inoculation: Xcc-SU monoculture, (**D**) 48 h after inoculation: *B. velezensis* monoculture, (**E**) 12 h after inoculation: dual culture, (**F**) 48 h after inoculation: dual culture. The ratio of *B. velezensis* cells to Xcc-SU cells at the time of inoculation was 1:100.

Scanning electron microscopy was used to examine the effect of extracellular metabolites on the cell morphology of Xcc-SU and *B. velezensis*. No change in the cellular form of *B. velezensis* occurred during cultivation in the dual culture with Xcc-SU (Figure 4E,F). *Bacillus* formed motile rod-shaped cells with a cell width of 0.60 ± 0.04 μm and length of 2.56 ± 0.67 μm. The Xcc-SU monoculture control showed regular, full, smooth rod-shaped cells whose dimensions were 0.44 ± 0.07 μm (width) and 1.31 ± 0.22 μm (length) during the whole experiment. In contrast, Xcc-SU morphology was significantly changed in the dual culture over time. The loss of viability of Xcc-SU in the dual culture (see Figure 2) was paralleled by shrinkage and distortion of the cells, accompanied by a wrinkled surface. This damaged the outer wall compared to the monoculture control (Figure 4A,C). After 48 h, a complete collapse of some Xcc cells was observed (Figure 4F). Evidently, the cell surface of Xcc-SU and the cell membrane were damaged by exposure to the antagonist as ruptures of the Xcc cells could be seen accompanied by leakage of cytoplasm (Figure 4F). Similar cell-perturbing effects were observed when *X. axonopodis* pv. *vesicatoria* and *P. aeruginosa* were exposed to fengycin from *B. amyloliquefaciens* [27] or when *Brachyspira hyodysenteriae* was exposed to surfactin at 10–250 μg/mL [20]. Bacillomycin D-like compounds isolated from *B. amyloliquefaciens* HAB-2 were also reported to produce a roughening of the cell surface and membrane damage in *B. pseudomallei* cells [32], similar to what we observed (Figure 4F). Such effects of lipopeptides were also reported in fungal organisms, e.g., *F. graminearum* and *A. flavus* [22,29,63]. The ability of lipopeptides to form pores in the membrane structure and to cause lysis of erythrocytes was demonstrated [5,64,65]. It is evident that the combined presence of surfactin, fengycin, and bacillomycin produced by *B. velezensis* caused significant cell surface damage in Xcc-SU, which resulted in the rapid elimination of Xcc-SU from the dual culture. A similar combined effect of several lipopeptides was observed in *B. subtilis*, active in the biological control of some fungal phytopathogens [24,26], and in *B. amyloliquefaciens* disrupting the structure of the conidia and mycelium of *Botryosphaeria dothidea* causing peach gummosis [66].

## 4. Conclusions

A detailed analysis of the antagonistic interaction of *B. velezensis* FZB42 and the phytopathogen *Xanthomononas campestris* pv. *campestris* under in vitro conditions was carried out in order to monitor the kinetics of Xcc death overtime and quantify major lipopeptides involved in the biological control of the phytopathogen. Novel findings included the demonstration of a combined effect of surfactin, fengycin, and bacillomycin, causing the death of the Xcc population, even when the lipopetides were present at relatively low levels. The antagonistic interaction was investigated using a new Xcc strain freshly isolated from a cabbage field struck with black rot infection. The mechanism of killing was documented by visualizing the attack of the bacterial cell wall and cytoplasmic membrane using SEM. The results confirmed the remarkable potential of *B. velezensis* strain FZB42 for biological control of phytopathogens, including Xcc, a causative agent of black rot of cruciferous vegetables.

## Data Availability

The obtained 16S rRNA gene sequence of the strain Xcc-SU was submitted in GenBank under accession number MZ182354.

## Supplementary Materials

The following are available online at https://www.mdpi.com/article/10.3390/microorganisms9071410/s1, Figure S1: Extracted ion chromatograms of bacillomycin D ([M+H]^+^ = 1031.541 *m/z*, 1A), surfactin ([M+H]^+^ = 1036.690 *m/z*, 2A), bacilibactin ([M+H]^+^ = 883.263 *m/z*, 3A), and fengycin A ([M+2H]^2+^ = 732.405 *m/z*, 4A) in a particular retention times. Table S1: Metabolites detected in dual culture of *B. velezensis* FZB42 and Xcc-SU.
