# Supplementary material for: Killing Effect of Bacillus velezensis FZB42 on a Xanthomonas campestris pv. Campestris (Xcc) Strain Newly Isolated from Cabbage Brassica oleracea Convar. Capitata (L.): A Metabolomic Study
Source: Microorganisms. 2021 Jun 29;9(7):1410. doi: 10.3390/microorganisms9071410 (PMC8303752; doi:10.3390/microorganisms9071410)
Supplement: Supplementary file 1 [file microorganisms-09-01410-s001.zip › microorganisms-1256696-supplementary.pdf]

Supplementary file

Killing effect of *Bacillus velezensis* FZB42 on a *Xanthomonas campestris* pv. *campestris* (Xcc) strain newly isolated from cabbage *Brassica oleracea* convar. *capitata* (L.): a metabolomic study

Hynek Mácha[1,2], Helena Marešová[1], Tereza Juříková[1], Magdaléna Švecová[1], Oldřich Benada[1], Anton Škríba[1], Miroslav Baránek[3], Čeněk Novotný[1], Andrea Palyzová[1*]

[1] Institute of Microbiology of the Czech Academy of Sciences, Vídeňská 1083, 142 20 Prague, Czech Republic; palyzova@biomed.cas.cz (A.P.); hynek.macha@biomed.cas.cz (H.M.); maresova@biomed.cas.cz (H.M.); tereza.jurikova@biomed.cas.cz (T.J.); majdulas@seznam.cz (M.Š); benada@biomed.cas.cz (O.B.) anton.skriba@biomed.cas.cz (A.Š.); baranek@mendelu.cz (M.B.); novotny@biomed.cas.cz (Č.N.); palyzova@biomed.cas.cz (A.P.)

[2] Department of Analytical Chemistry, Faculty of Science, Palacký University, 17. Listopadu 12, 771 46 Olomouc, Czech Republic; hynek.macha@biomed.cas.cz (H.M.)

[3] Faculty of Horticulture-Mendeleum, Mendel University, Valtická 337, 69144 Lednice, Czech Republic; baranek@mendelu.cz (M.B.)

* Correspondence: palyzova@biomed.cas.cz (A.P.)

Contents **Page**

Table S1: Metabolites detected in dual culture of *B. velezensis* FZB42 and Xcc-SU 2

Figure S1: Extracted ion chromatograms of bacillomycin D ([M+H]$^+$=1031.541 *m/z*, 1A), surfactin ([M+H]$^+$ =1036.690 *m/z*, 2A), bacilibactin ([M+H]$^+$ =883.263 *m/z*, 3A), and fengycin A ([M+2H]$^{2+}$ = 732.405 *m/z*, 4A) in a particular retention times. Corresponding fragmentation spectra of selected parent ions of bacillomycin D (1B), surfactin (2B), bacilibactin (3B), and fengycin A (4B) are in agreement with the literature [41,49]. 3

Abbreviations 4

References 4

Table S1: Metabolites detected in dual culture of *B. velezensis* FZB42 and Xcc-SU

metabolite	ion type	measured m/z	Molecular formula	Calculated m/z	LC-MS spectra
Bacillomycin D (C12)	$[M+H]^+$	1003.506	$C_{46}H_{70}N_{10}O_{15}$	1003.509	
Bacillomycin D (C13)	$[M+H]^+$	1017.514	$C_{47}H_{72}N_{10}O_{15}$	1017.525	
Bacillomycin D (C14)	$[M+H]^+$	1031.548	$C_{48}H_{74}N_{10}O_{15}$	1031.541	Fig.S1A,B
	$[M+Na]^+$	1053.523	$C_{48}H_{74}N_{10}O_{15}$	1053.523	
Bacillomycin D (C15)	$[M+H]^+$	1045.551	$C_{49}H_{76}N_{10}O_{15}$	1045.556	
	$[M+Na]^+$	1067.546	$C_{49}H_{76}N_{10}O_{15}$	1067.538	
Bacillomycin D (C16)	$[M+H]^+$	1059.564	$C_{50}H_{78}N_{10}O_{15}$	1059.572	
	$[M+Na]^+$	1081.547	$C_{50}H_{78}N_{10}O_{15}$	1081.554	
Bacillomycin D (C17)	$[M+H]^+$	1073.584	$C_{51}H_{80}N_{10}O_{15}$	1073.588	
Surfactin (C50)	$[M+H]^+$	994.639	$C_{50}H_{87}N_7O_{13}$	994.643	
	$[M+Na]^+$	1016.623	$C_{50}H_{87}N_7O_{13}$	1016.625	
Surfactin (C51)	$[M+H]^+$	1008.654	$C_{51}H_{89}N_7O_{13}$	1008.659	
	$[M+Na]^+$	1030.637	$C_{51}H_{89}N_7O_{13}$	1030.641	
Surfactin (C52)	$[M+H]^+$	1022.669	$C_{52}H_{91}N_7O_{13}$	1022.675	
	$[M+Na]^+$	1044.648	$C_{52}H_{91}N_7O_{13}$	1044.657	
Surfactin (C53)	$[M+H]^+$	1036.697	$C_{53}H_{93}N_7O_{13}$	1036.690	Fig.S2A,B
	$[M+Na]^+$	1058.669	$C_{53}H_{93}N_7O_{13}$	1058.672	
Bacillibactin	$[M+H]^+$	883.263	$C_{39}H_{42}N_6O_{18}$	883.263	Fig.S3A,B
	$[M+Na]^+$	905.244	$C_{39}H_{42}N_6O_{18}$	905.245	
Fengycin A (C15-un-saturated)	$[M+2H]^{2+}$	724.411	$C_{71}H_{106}N_{12}O_{20}$	724.390	
Fengycin A (C15)	$[M+2H]^{2+}$	725.398	$C_{71}H_{108}N_{12}O_{20}$	725.397	
Fengycin A (C16-un-saturated)	$[M+2H]^{2+}$	731.403	$C_{72}H_{108}N_{12}O_{20}$	731.397	
Fengycin A (C16)	$[M+2H]^{2+}$	732.406	$C_{72}H_{110}N_{12}O_{20}$	732.405	Fig.S4A,B
Fengycin A (C17-un-saturated)	$[M+2H]^{2+}$	738.428	$C_{73}H_{110}N_{12}O_{20}$	738.405	
Fengycin A (C17)	$[M+2H]^{2+}$	739.412	$C_{73}H_{112}N_{12}O_{20}$	739.413	
Fengycin A (C18)	$[M+2H]^{2+}$	746.428	$C_{74}H_{114}N_{12}O_{20}$	746.421	
Fengycin A (C19)	$[M+2H]^{2+}$	753.429	$C_{75}H_{116}N_{12}O_{20}$	753.429	
Fengycin A (C20)	$[M+2H]^{2+}$	760.427	$C_{76}H_{118}N_{12}O_{20}$	760.437	
Fengycin A (C21)	$[M+2H]^{2+}$	767.433	$C_{77}H_{120}N_{12}O_{20}$	767.444	
Fengycin A (C22)	$[M+2H]^{2+}$	774.435	$C_{78}H_{122}N_{12}O_{20}$	774.452	
Fengycin A (C23)	$[M+2H]^{2+}$	781.444	$C_{79}H_{124}N_{12}O_{20}$	781.460	
Fengycin A (C24)	$[M+2H]^{2+}$	788.447	$C_{80}H_{126}N_{12}O_{20}$	788.468	

Figure S1: Extracted ion chromatograms of bacillomycin D ([M+H]+=1031.541 *m/z*, 1A), surfactin ([M+H]+ =1036.690 *m/z*, 2A), bacilibactin ([M+H]+ =883.263 *m/z*, 3A), and fengycin A ([M+2H]2+ = 732.405 *m/z*, 4A) at particular retention times. Corresponding fragmentation spectra of selected parent ions of bacillomycin D (1B), surfactin (2B), bacilibactin (3B), and fengycin A (4B) are in agreement with the literature [41,49].

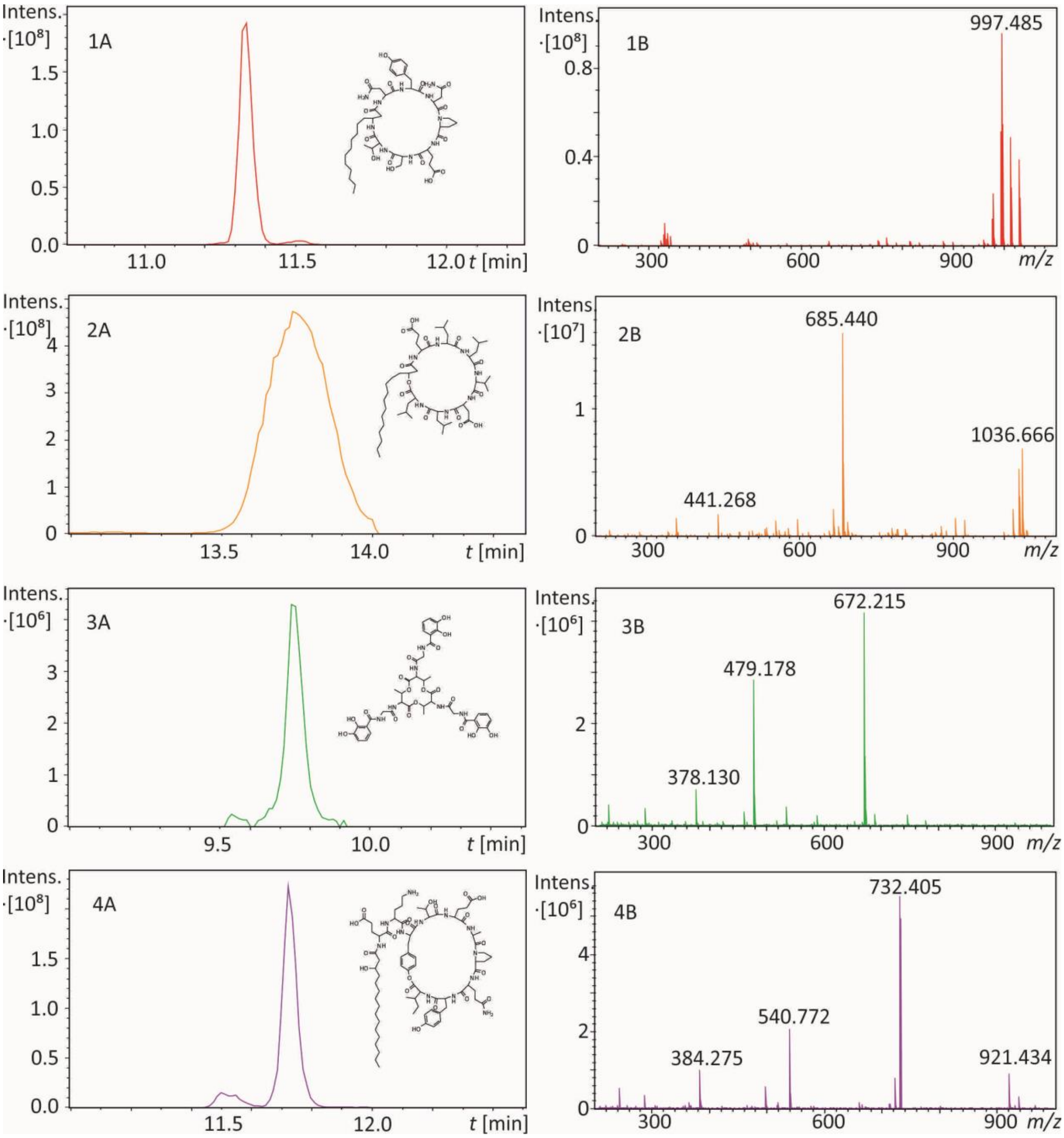

Abbreviations: BH, Bushnel-Haas medium; CBS, concentric backscatter detector; CFU, colony forming unit; ESI, electrospray ionization; ETD, Everhart-Thornley Detector; HPLC, high-performance liquid chromatography; LB, Luria-Bertani broth; LC-MS, liquid chromatography-mass spectrometry; LOD, limit of detection; LOQ, limit of quantitation; M9, mineral medium; M9TE, M9 mineral medium supplemented with trace elements; MS, mass spectrometry; PCR, polymerase chain reaction; SEM, scanning electron microscopy; TLD, through-the-lens detector; Xcc, *Xanthomonas campestris* pv. *campestris*

References

41. Palyzová, A.; Svobodová, K.; Sokolová, L.; Novák, J.; Novotný, Č. Metabolic profiling of *Fusarium oxysporum* f. sp. *conglutinans* race 2 in dual cultures with biocontrol agents *Bacillus amyloliquefaciens*, *Pseudomonas aeruginosa*, and *Trichoderma harzianum*. Folia Microbiol. **2019**, 64, 779-787 doi: 10.1007/s12223-019-00690-7

67. Chen, Z.; Wu, Q.; Wang, L.; Chen, S.; Lin, L.; Wang, H.; Xu, Y. Identification and quantification of surfactin, a nonvolatile lipopeptide in Moutai liquor. Int. J. Food Prop. **2020**, 23, 189-198.
